# Effect of parental touch on relieving acute procedural pain in neonates and parental anxiety (Petal): a multicentre randomised controlled trial

**DOI:** 10.1016/S2352-4642(23)00340-1

**Published:** 2024-02-16

**Authors:** Annalisa G V Hauck, Marianne van der Vaart, Eleri Adams, Luke Baxter, Aomesh Bhatt, Daniel Crankshaw, Amraj Dhami, Ria Evans Fry, Marina B O Freire, Caroline Hartley, Roshni C Mansfield, Simon Marchant, Vaneesha Monk, Fiona Moultrie, Mariska Peck, Shellie Robinson, Jean Yong, Ravi Poorun, Maria M Cobo, Rebeccah Slater

**Affiliations:** 1 Department of Paediatrics, University of Oxford, Oxford, UK; 2 Newborn Care Unit, John Radcliffe Hospital, Oxford University Hospitals NHS Foundation Trust, Oxford, UK; 3 University of Exeter Medical School, University of Exeter, Exeter, UK; 4 Department of Paediatrics, Royal Devon University Healthcare NHS Foundation Trust, Exeter, UK; 5 Universidad San Francisco de Quito USFQ, Colegio de Ciencias Biologicas y Ambientales, Quito, Ecuador

**Keywords:** Nerve fibres, unmyelinated, STAI, anxiety, event related potential, gentle touch

## Abstract

**Background:**

Touch interventions such as massage and skin-to-skin contact relieve neonatal pain. The Parental touch trial (Petal) aim was to assess whether parental stroking of their baby prior to a clinically-required heel lance, at a speed of approximately 3 cm/s to optimally activate C-tactile (CT) nerve fibres, provides effective pain relief.

**Methods:**

Petal is a multicentre, randomised, parallel-group interventional trial. Neonates without neurological abnormalities who were born at 35 weeks gestational age or above and required a blood test via a heel lance in the first week of life were randomised to receiving parental touch for 10 s either before (intervention group) or after (control group) the clinically-required heel lance. The primary outcome measure was the magnitude of noxious-evoked brain activity in response to the heel lance measured with electroencephalography (EEG). Secondary outcome measures were (i) Premature Infant Pain Profile-Revised (PIPP-R) score; (ii) development of tachycardia; and (iii) parental anxiety score. The trial is registered on ISRCTN (ISRCTN14135962) and ClinicalTrials.gov (NCT04901611).

**Findings:**

Between 1^st^ September 2021, and 7^th^ February 2023, 56 neonates were allocated to the intervention and 56 to the control group. The primary outcome did not differ significantly between groups, with a mean difference of -0·11 (smaller in intervention group, SD 0·77, 95% CI=[-0·42,0·20], p-value 0·38, n=82). No significant difference was observed across secondary outcomes: (i) the PIPP-R difference in means was 1.10 (greater in intervention group, 95% CI=[-0·42,2·61], p=0·15, n=100), (ii) the odds ratio of becoming tachycardic was 2·08 (greater in the intervention group, 95% CI=[0·46,9·46], p=0·34, n=105), (iii) the difference in parental State-Trait Anxiety Inventory-State was -0·44 (greater in control group, 95% CI=[-2·91,2·02], p=0·72, n=106). One serious adverse event occurred; this was not considered to be related to the study.

**Interpretation:**

Parental stroking delivered at an optimal speed to activate CT fibres for a duration of 10 s prior to the painful procedure did not significantly change neonates’ magnitude of pain-related brain activity, PIPP-R score, or development of tachycardia. The trial highlighted the challenge of translating an experimental researcher-led tactile intervention into a parent-led approach, and the value of involving parents in their baby’s pain management.

**Funding:**

This work is supported by a Wellcome Senior Fellowship awarded to Rebeccah Slater (grant number 207457/Z/17/Z) and by Bliss (a UK charity) via a research grant.

## Introduction

Newborn babies undergo clinically necessary painful procedures in their first days of life,^1^ for example to take blood samples to check for serious health conditions. Effective pain management is imperative, and babies could benefit from pain-relieving interventions that are provided by their parents. While pharmacological interventions can be used to treat pain, there are challenges in determining the optimal dosages, and finding the balance between the need for pain relief and potential side effects. Non-pharmacological interventions such as massage^2,3^ and “skin-to-skin” contact^4^, are used to relieve neonatal pain.^5^ However, given the subjective nature of pain and the pre-verbal nature of neonates, determining the effectiveness of these pain-relieving interventions is challenging. This is exacerbated by the reliance on pain assessment approaches that involve subjective judgements. An alternative approach is to use electroencephalography (EEG) as an objective endpoint in clinical trials of analgesics to measure noxious-evoked brain activity.^6,7^

Brain-derived methods have been used to investigate the impact of maternal skin-to-skin care on premature neonates during clinical procedures.^8–11^ In one randomised controlled trial, a group of 73 premature neonates who received maternal skin-to-skin contact (kangaroo care) prior to a heel lance had a lower heart rate and Premature Infant Pain Profile (PIPP) score, and a higher level of oxygen saturation and regional cerebral tissue oxygenation saturation (rcSO2) compared with a control group.^11^ In a second prospective crossover study in 10 premature neonates, babies held in skin-to-skin contact with their mothers during venepuncture had significantly smaller increases in cerebral oxyhaemoglobin compared to when they were in their crib or incubator.^9^ A third prospective cross-sectional study recorded EEG during a clinically-required heel lance procedure in 18 premature and term-born neonates. This study reported that maternal skin-to-skin contact led to a significantly reduced noxious event-related potential compared to being held while wearing clothes. There was, however, no significant difference between the neonates who received skin-to-skin contact and the neonates who were swaddled or nested while receiving individualised care.^8^ This result was interpreted as a reflection of the success of the individualized and developmentally sensitive care, rather than a failure of skin-to-skin care in dampening noxious-related activity.^8^

While the efficacy of these non-pharmacological approaches is evidence based, they are less frequently used in maternal and neonatal units compared with the administration of sucrose, where the availability of specific guidelines regarding the optimal dose, timing and route of administration allows for easier implementation.^12^ A better understanding of the mechanisms that underpin the benefits of parental touch could be used to simplify and optimise pain management guidelines. One possible mechanism underpinning the analgesic efficacy of multiple touch-based interventions is the activation of C-tactile (CT) fibres, which are reported to encode affective dimensions of touch.^13^ CT fibres are unmyelinated, slow conducting afferents found in hairy skin,^13,14^ which respond optimally to gentle touch when applied in a typical caressing motion at a speed of 1–10 cm/s at skin temperature.^15^ In adults, touch at CT-optimal speed is perceived as pleasant, and can decrease verbal pain reports and noxious-evoked brain activity.^16–18^ A calming effect – observed as a reduction in heart rate – has also been reported in neonates and infants stroked at these speeds.^19–21^ Based on this evidence, we explored the effect of experimenter-led soft brushing of the skin at CT-optimal speeds in term neonates. Brushing the babies’ skin was associated with a reduction of approximately 40% in noxious-evoked brain activity in response to both experimental and clinically-required noxious procedures.^22,23^

In the Parental touch trial (Petal), we build on this evidence. We tested the hypothesis that parental stroking at CT-optimal speed prior to a clinically-required heel lance reduces noxious-evoked brain activity compared with standard care. In contrast to the pilot studies, where the baby’s skin was stroked using a calibrated brush by an experienced researcher, here we wanted to establish whether gentle touch delivered by a parent evoked a similar reduction in noxious-evoked brain activity.

Given the value of using brain-derived approaches to assess the efficacy of analgesia in neonates,^24,25^ we selected noxious-evoked brain activity as the primary outcome measure. Secondary outcomes included a clinical pain score (Premature Infant Pain Profile – Revised; PIPP-R) and the occurrence of tachycardia. Parental touch behaviours are instinctive and can benefit both babies and parents during painful procedures,^2^ however watching a painful procedure can cause parental anxiety and emotional distress.^26^ Therefore, parental anxiety was evaluated as a secondary outcome. In addition, as part of an exploratory study reported elsewhere,^27^ we explored parental experiences during the procedures and overall parental satisfaction related to trial participation.

## Methods

### Study design and participants

Petal is a multicentre, randomised, two-arm, parallel, controlled, superiority trial. Participants were recruited from two centres: the John Radcliffe Hospital (Oxford University Hospitals NHS Foundation Trust, UK) and the Royal Devon and Exeter Hospital (Royal Devon University Healthcare NHS Foundation Trust, UK).

Neonates were assessed for eligibility at the time of recruitment, and reassessed at the time of randomisation. Neonates considered eligible for inclusion were (i) born at 35 weeks gestational age or more, (ii) less than eight postnatal days and (iii) required a heel lance for clinical blood sampling. Exclusion criteria were (i) hypoxic ischaemic encephalopathy, (ii) intraventricular haemorrhage above grade II, (iii) receipt of analgesics or sedatives in the 24 hours prior to the study, (iv) born with a congenital malformation or genetic condition known to affect neurological development, or (v) born to a mother with a history of substance abuse.

Parents were verbally informed about the study and given written information via a participant information leaflet (Appendix p 8). Written informed parental consent was obtained for all neonates (sample consent form in Appendix p 8). The study received approval from the London-South East Research Ethics Committee of the National Research Ethics Service (ref: 21/LO/0523) and conformed to the Declaration of Helsinki and Good Clinical Practice standards. A full description of the trial protocol has been published.^28^

### Randomisation and masking

Neonates were randomised to receive parental tactile stimulation (10 s of parental stroking of the baby’s lower leg) either before the heel lance (intervention group), or after the heel lance (control group). Allocation ratio to the intervention or control group was 1:1 and a minimisation algorithm featuring a probability-based randomisation element was used to balance demographic variables between the groups. The five minimisation criteria were (i) gestational age at birth, (ii) postnatal age at study, (iii) sex, (iv) site and (v) primary reason for blood test. Randomisation was managed centrally at the Oxford site using a web-based system provided by Sealed Envelope Ltd (London, UK). The web-based facility did not allow insight into the next participant’s allocation to ensure allocation concealment.

As parents were responsible for delivering the intervention, the research team informed the parents of their baby’s group allocation prior to the heel lance. To ensure that group allocation did not impact the baseline State Trait Anxiety Inventory (STAI), parents completed the initial questionnaire before group allocation disclosure.

### Procedures

Each baby was studied on a single occasion. Continuous vital signs monitoring (electrocardiogram and pulse oximetry) started approximately 30 minutes before the heel lance and continued for 30 minutes after. Neonatal EEG was recorded for at least 10 minutes before and after the heel lance. Eight EEG recording electrodes were positioned on the scalp at Cz, CPz, C3, C4, FCz, T3, T4 and Oz according to the modified international 10–20 system. Reference and ground electrodes were placed at Fz and Fpz, respectively. Comfort measures, which included swaddling and non-nutritive sucking, were offered to all neonates independent of group allocation, in line with local practice guidelines. Before the heel lance, a sham heel lance was performed to assess the neonate’s response to a stimulus identical to the lance, but without the noxious component – for the sham procedure the lancet was rotated by 90° before being placed against the foot, such that the blade did not touch the baby. During both sham procedure and clinical heel lance, a video of the baby’s facial expressions was recorded. The videos were used to categorise the behavioural state of the babies prior to the heel lance using the groups described in the PIPP-R score. While the PIPP-R score uses four behavioural states, in this analysis we grouped the categories as either ‘awake’ or ‘asleep’. The sham heel lance and clinical heel lance were time-locked to the EEG recordings using an automated detection interface.^29^ The start and end of the parental stroking were time-locked by the researcher pressing a button to event mark the recordings. Events were time-locked to the vital signs recordings via an automated detection interface (Oxford)^30^ or by a researcher manually annotating recordings (Exeter). The timings of the sham heel lance and heel lance were identifiable in the video recordings by an LED-light that was activated by the clinical researcher at the time of the procedures.

The EEG recordings were used to assess the magnitude of noxious-evoked brain activity, videos were used to calculate the PIPP-R score,^31^ and vital signs were used to calculate the occurrence of tachycardia in response to the heel lance and to calculate the heart rate and oxygen saturation components of the PIPP-R score.^31^ An overview of the trial procedures is provided in the Appendix (p 9).

In the intervention group, the parent stroked their baby’s lower leg just before the heel lance, while in the control group the parent stroked their baby’s lower leg at least 30 s after the heel lance at a time considered appropriate by the clinician performing the heel lance, to ensure blood collection was not disrupted. Active collection of the blood sample, which can involve applying gentle pressure to the baby’s foot to collect an adequate quantity of blood, commenced at least 30 s after the heel lance. This ensured that the blood collection process did not impact the PIPP-R score.

Parents stroked their baby’s leg for 10 s at approximately 3 cm/s. This duration was chosen to match the duration of the stroking intervention used in two pilot studies, where the intervention significantly reduced the magnitude of the noxious-evoked brain activity.^22,23^ The stroking was guided by a computer animation displayed on a screen. The animation showed a 3 s countdown timer to identify the start of the stroking motion. This was followed by a progress bar that extended 3 times at a speed of 3 cm/s over a 10 cm distance for a duration of 10 s. The mode of delivery (parental hand), duration (10 s), approximate speed (3 cm/s), number of stroking motions (3 times), location (neonate’s lower leg ipsilateral to the heel lance) and direction of stroking (along the limb) did not differ between trial arms. Parents demonstrated to the researchers that they knew how to follow the animation and stroke their baby according to the trial protocol before commencing the stroking intervention.

At the start of each test occasion, parents answered both the Trait and State components of the STAI.^32^ After the heel lance, parents completed the STAI-State (STAI-S) for a second time, and completed the 4-point distress questionnaire, which asked about their feelings during the heel lance.^32^ In addition, they completed an anonymous survey to describe their motivations for taking part in the trial, and their experiences and emotions related to their trial involvement.^27^

### Outcomes

The primary outcome measure was the magnitude of noxious-evoked brain activity during a clinically-required heel lance. Noxious-evoked brain activity was quantified using a noxious neurodynamic response function (n-NRF), whereby a fixed-shape waveform is fitted to each neonate’s EEG data, in a process akin to the use of a haemodynamic response function in fMRI studies.^33^ The n-NRF has been developed and validated to quantify the characteristic waveform evoked by noxious stimuli in neonates.^6^ It has been used to quantify the magnitude of noxious-evoked brain activity following a heel lance^6,7,22,23,34^ and to assess efficacy of analgesic interventions in neonates,^6,7^ including researcher-led tactile stroking.^22,23^ The magnitude of the n-NRF is measured approximately 400-700 ms post-stimulation by linearly regressing the n-NRF onto EEG data recorded in this time-window. The regression coefficient is the magnitude of noxious-evoked brain activity. A detailed description of the EEG analysis methods is in the Appendix (pp 9-10).

Secondary outcomes were (i) PIPP-R^31^ scores following the heel lance; (ii) the development of neonatal tachycardia following the heel lance;^35,36^ and (iii) parental anxiety scores after the heel lance measured with the STAI-S.^32^ The heel lance was considered to cause tachycardia if the neonate’s heart rate was not above 160 bpm in the 15 seconds baseline period but greater than or equal to 160 bpm in the 30 s period following the heel lance.^35,36^ STAI-S scores were calculated according to the STAI manual.^32^ Twenty percent of total PIPP-R scores in response to the heel lance were rescored to assess intra- and inter-rater reliability with intraclass correlation coefficient (ICC). Inter-rater ICC was 0·98 (95% CI=[0·95,0·99]), intra-rater ICC was 0·99 (95% CI=[0·98,0·99]).

Quantification of the n-NRF magnitude (primary outcome measure), PIPP-R vital signs components and tachycardia outcome (secondary outcomes) was performed using automated scripts on blinded data and did not involve any subjective assessment. Subjective quality assessment of the EEG and vital signs data for artefact detection was performed by two blinded investigators, with any discrepancies in assessment resolved by discussion. Facial expression components of the PIPP-R were scored by researchers who had not been involved in the specific test occasion and who were blind to the procedure (sham heel lance or heel lance) and trial arm allocation. STAI-S scores were entered into an electronic database and the full score computed according to the STAI-S user guide.^32^ Further details on outcome assessment and blinding are in the SAP, which was finalised prior to unblinding the trial data.^37^

### Statistical analysis

Based on previous research,^22,23^ we consider a 40% reduction in the intervention group to be clinically significant. For sample size calculation, the mean n-NRF magnitude evoked by heel lancing in the control group is estimated to be 1*·*07 with a standard deviation (SD) of 0*·*66. Thus, the intervention arm heel lance-evoked mean n-NRF magnitude is set at 0*·*642 and SD is 0*·*66. With 90% power, a two-sided 5% significance level, and an allocation ratio of 1:1, we estimated a sample size of n=102 neonates. Allowing for data loss of approximately 10%, the final sample size is n=112.

Parental compliance in delivering the intervention was assessed qualitatively by the researcher present during the study and, in the intervention group, by calculating the time delay between the stroking intervention and the heel lance. If parental stroking started more than 45 s prior to the heel lance, this was considered as non-compliance with regard to assessing the neonates’ outcomes (noxious-evoked brain activity, tachycardia and PIPP-R), since the timing of the intervention has a direct and established impact on these outcome measures, as the analgesic effect of stroking has been reported in adults to diminish over time.^17^

To study the effect of the stroking intervention, we estimated the per protocol effect, which is the effect of the intervention in those who adhered to (complied with) the intervention requirements. Simply excluding non-compliers from the analysis (naïve per protocol analysis) can lead to biased estimates. We thus performed complier average causal effect (CACE) analysis on the full analysis set to appropriately account for non-compliance in an unbiased manner.^38^ The full analysis set includes all randomised patients with a measured outcome, and CACE analysis isolates the per protocol effect through instrumental variable analysis using the two-stage least squares approach.^39^ For completeness, we also performed an exploratory analysis of the intention-to-treat (ITT) effect on the full analysis set, which estimates the overall effect of intervention effectiveness taking into account the impact of non-compliance (Appendix pp 10, 18).

Regarding the statistical models used, the primary outcome measure, which was the magnitude of the n-NRF, and the secondary outcomes PIPP-R and parental STAI-S were compared between the two groups using multiple linear regression analysis. The development of tachycardia (binary secondary outcome) was compared between the groups using a logistic regression. In the regression models, the group allocation variable was adjusted for the five minimisation variables (gestational age, postnatal age, site, sex and primary reason for blood test). For the analysis of the secondary STAI-S outcome the group allocation variable was also adjusted for the STAI-S at baseline (i.e., before the heel lance). Based on regression model assumption testing results (as outlined in the Appendix), we performed robust linear regressions and report non-parametric p-values derived using permutation testing (Appendix pp 10-11).

The significance level for the primary outcome was set at 0·05. An overall alpha level of 0·05 was shared among the three secondary outcomes and adjusted for multiple comparisons using the Holm method. We report mean (SD) effect sizes and 95% CI for all outcomes. As specified in the SAP, the effect on the tachycardia outcome is reported as odds ratio. For completeness, the risk ratio is also reported (Appendix pp 11,18). All statistical analyses were performed in R (version 4·2·2 or newer) or MATLAB (Mathworks, version 9·14, R2023a).

The approaches to assess data loss are described in full in the SAP.^37^ Serious adverse events (SAE) definitions are reported in the full trial protocol. Besides SAEs, no pre-specified adverse events were provided in the trial protocol. The trial is registered with ISRCTN (identifier ISRCTN14135962) and ClinicalTrials.gov (identifier NCT04901611). The trial is reported according to CONSORT 2010 guidelines, and a checklist is available (Appendix pp 11-13).

### Adherence to trial procedures

Data quality and adherence to trial procedures was assessed by the Project Management Group (PMG). PMG researchers blinded to the group allocation performed periodic data quality checks. The PMG held regular group meetings at the Oxford site, visited the Exeter site, created and ensured adherence to internal guidance sheets and hosted regular training and problem-solving sessions.

### Role of the funding source

The study funders had no role in study design, data collection, data analysis, data interpretation, or writing of the report.

## Results

**Figure 1:**
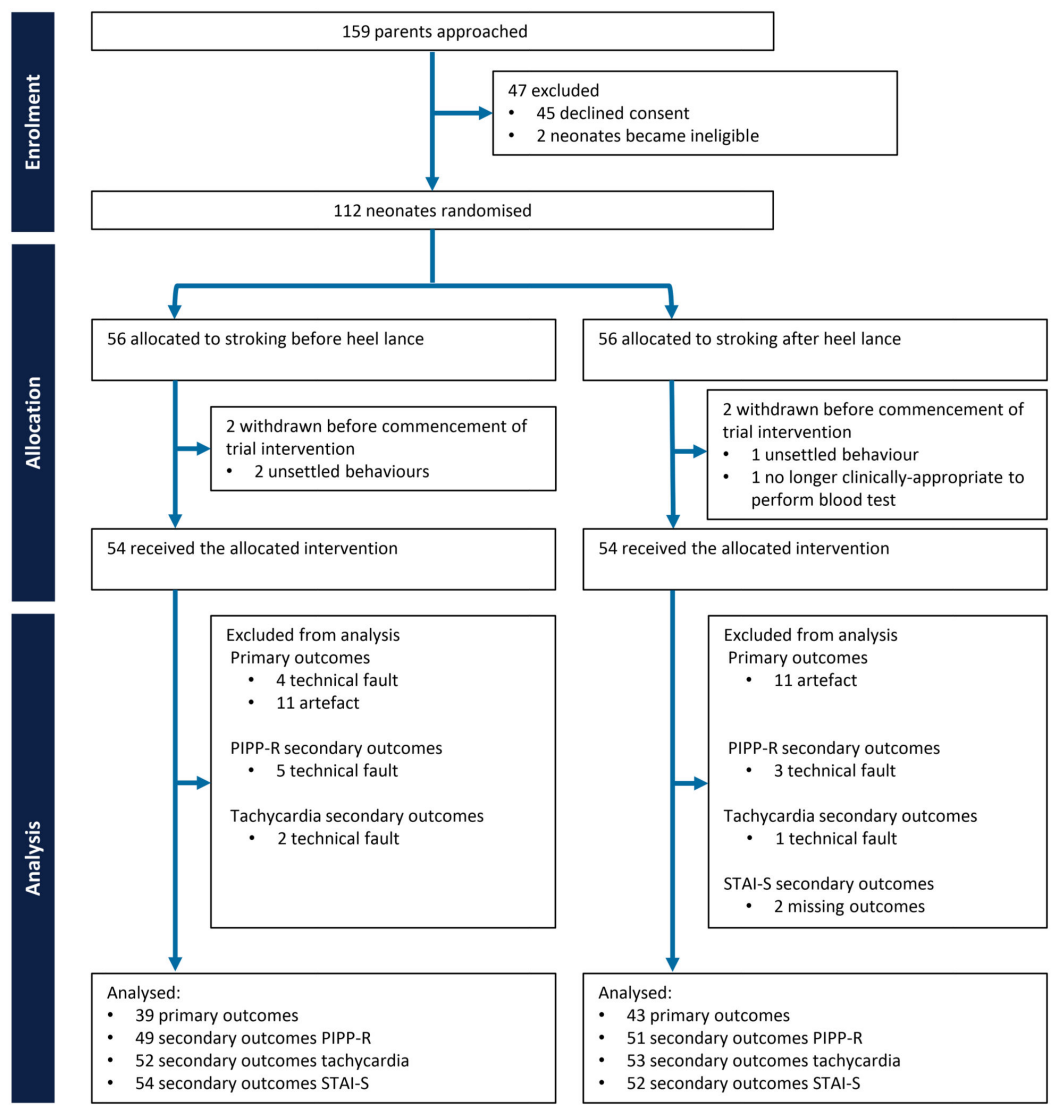
Trial profile. PIPP-R=premature infant pain profile-revised. STAI-S=State Trait Anxiety Inventory-State. Artefacts that led to rejection of epochs for primary outcome measure analysis were either movement or electrical artefacts.

Between 1 September 2021 and 7 February 2023, 112 neonates were randomised to either an intervention group (parental stroking before the heel lance, n=56) or a control group (parental stroking after the heel lance, n=56) (Figure 1).

Participant demographics and baseline clinical characteristics were balanced across groups (Table 1). Baseline characteristics of neonates included in the analysis of each outcome are available in the Appendix (pp 14-15). The median postmenstrual age of participants was 38·6 (IQR 37·2–40·3) weeks, and 68 (61%) were male. The median weight at birth was 3299 (IQR 2765–3767) g and the overall median number of painful procedures before the study was 4 (IQR 2–6). The primary reason for the blood test was a serum bilirubin check for jaundice for 54 (48%) neonates, followed by infection markers monitoring for management of potential sepsis in 33 (29%) neonates. Less common reasons were categorised as ‘other’ and included urea and haemoglobin measurements, newborn blood spot screening, and glucose monitoring. Mothers performed the stroking in 73 (65%) of the studies. Ethnicity of participants was recorded based on information in the medical notes and, where unclear, on parental report. Ethnicity was grouped according to the categories used in the census for England and Wales. 81% of the participants were White. The trial profile (Figure 1) lists the number of participants with an available outcome, i.e., the full analysis set. Parental stroking that commenced at least 45 seconds before the heel lance was considered as non-compliant with regards to assessing the neonate’s outcomes. After accounting for other sources of data loss (Figure 1), it impacted five primary outcomes, seven secondary PIPP-R outcomes, and eight secondary tachycardia outcomes.

**Figure 2:**
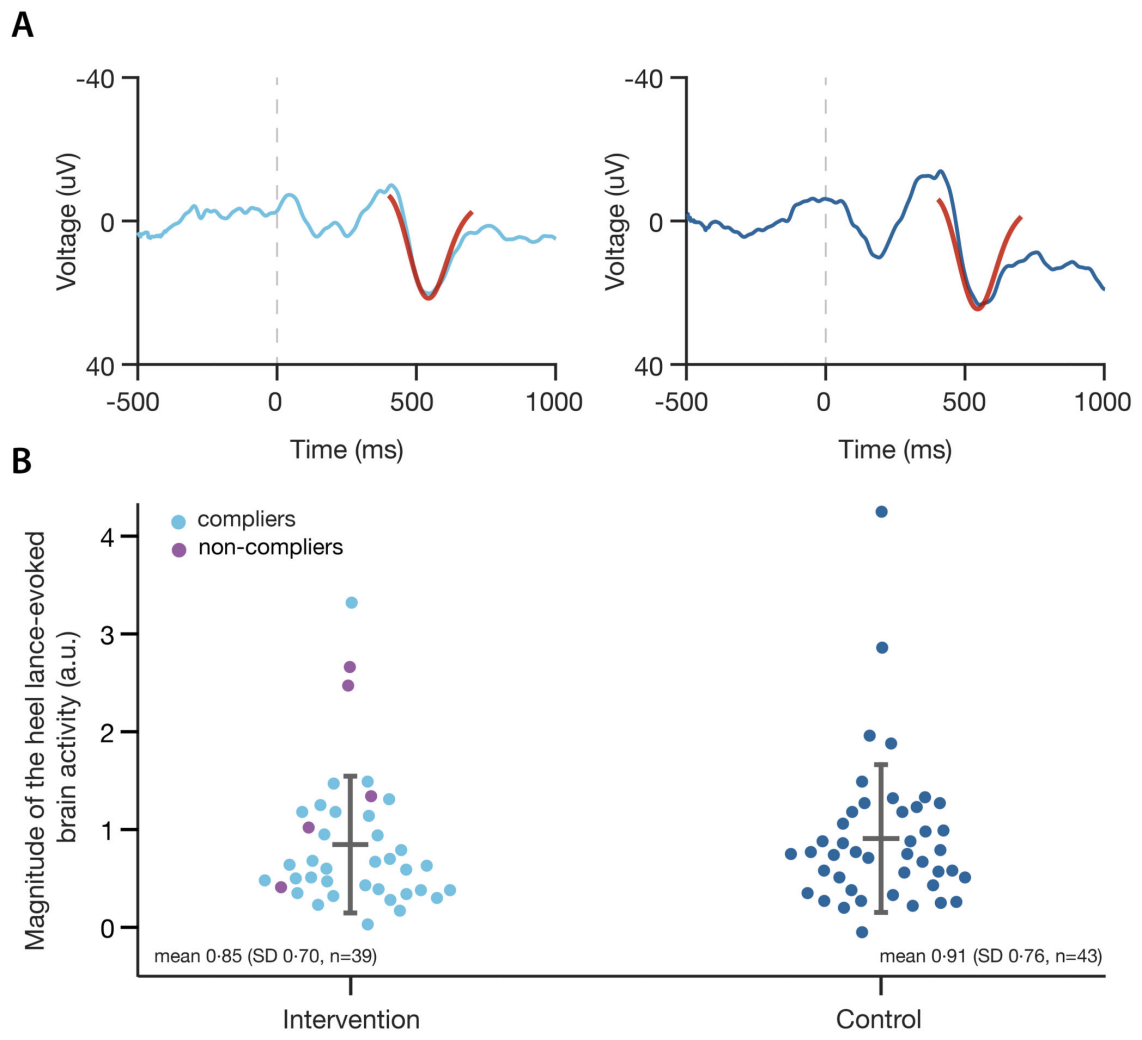
Primary outcome. Noxious-evoked brain activity in the intervention group and control group (A) Average EEG traces recorded at electrode Cz between 500 ms preceding and 1000 ms following the heel lance in the full analysis set grouped as randomised (n=82). EEG data is processed as described in the Appendix (pp 9-10), and the scaled noxious neurodynamic response function (n-NRF)^6^ is shown overlaid in red. (B) Magnitudes of the n-NRF. Each point in the two scatter plots represents the primary outcome measure for a single neonate; a.u.: arbitrary units. (Notes about the n-NRF: (i) the magnitude of noxious-evoked brain activity correlates with the intensity of the nociceptive input^40^; (ii) a scaled magnitude of 1 represents the expected response to a heel lance in term-aged infants^6^; (iii) non-pharmacological and pharmacological interventions reduce the magnitude of the response^6,22,23^ and (iv) a reduction of approximately 40% may be considered clinically meaningful – this is based on extrapolations from adult studies where a smaller reduction in the noxious-evoked potential is associated with significantly lower verbal pain scores.^18,22^)

The magnitude of the noxious-evoked brain activity, as quantified by the n-NRF – which was the primary outcome measure of the trial – did not significantly differ between the intervention and control groups (Figure 2). The difference in means was -0·11 (smaller in intervention group), SD 0·77, 95% CI=[-0·42,0·20], and p-value 0·38 (n=82). The median (IQR) time between the start of stroking and the heel lance was 16·9 (11·6–33·0) s (n=39).

**Figure 3:**
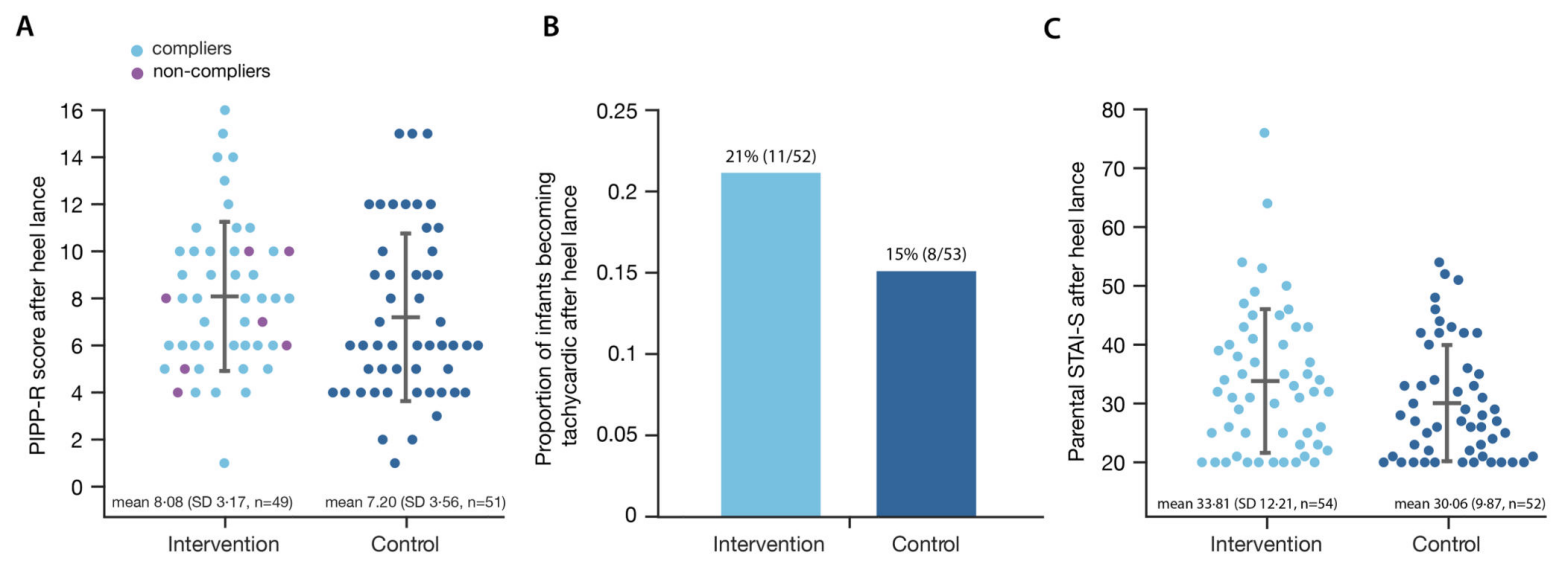
Secondary outcomes in the intervention and control groups (A) Total PIPP-R for each neonate. The PIPP-R score ranges from 0 to 21 and can be interpreted as no pain (0), mild (1–6), moderate (7–12) and severe (>12) pain (https://lab.research.sickkids.ca/stevens/pipp-r-module/). (B) Proportion of neonates who developed tachycardia after the heel lance. (C) Total STAI-S score after the heel lance for each parent who stroked their baby. The STAI-S score ranges from 20 to 80, with higher scores indicating higher anxiety levels. Mean STAI-S scores of approximately 35 are described in working adults,^32^ whereas scores of approximately 50 are reported by parents of neonates admitted to neonatal intensive care units in the UK and US.^26^ The mean difference between groups adjusted for baseline STAI-S and minimisation criteria was -0·44 (greater in control group, SD 6·85, n=106). In the scatter plots in (A) and (C), each dot represents the outcome measure for a single baby or their parent, respectively. This figure illustrates the full analysis set grouped as randomised.

None of the secondary outcomes differed significantly between the intervention and control groups (Figure 3). There were no differences between the groups’ PIPP-R following the heel lance. The difference in means between the two groups was 1.10 (greater in intervention group), SD 3·26, 95% CI=[-0.42,2.61], and p-value 0.15 (n=100, Figure 3A). The number of neonates who became tachycardic did not differ significantly between groups. The odds ratio (OR) was 2·08 (greater in the intervention group), 95% CI=[0·46,9·46], and p-value 0·34 (n=105, Figure 3B). For the parental anxiety STAI-S outcome, the mean difference between groups was -0·44 (greater in control group), SD 6·85, 95% CI=[-2·91,2·02], and p-value 0·72 (n=106, Figure 3C).

A data quality assessment was conducted to establish whether the outcome measures used to quantify noxious-evoked responses were significantly greater following the noxious heel lance as compared with the innocuous sham procedure. We confirmed that the magnitude of noxious-evoked brain activity and PIPP-R scores were significantly greater following the noxious stimulation compared with the non-noxious sham procedure (Appendix, pp 16-18). In addition, an exploratory analysis was conducted to assess the ITT effect. These ITT results are reported and lead to the same interpretation as the primary analysis (Appendix p 18).

Except for an instance where the parent switched from the correct (ipsilateral leg) to the contralateral leg between the first and second stroking movement, no protocol deviations occurred. As this baby was in the control group, this deviation does not affect the study. SAEs occurring during the trial were recorded and assessed by a senior clinician who considered their severity and whether there was a causal link between the events and trial participation. One SAE was recorded: this was unrelated to study participation and occurred in a baby randomised to the control group.

## Discussion

Various forms of dynamic tactile stimulation, including neonatal massage,^3^ skin-to-skin contact through the provision of kangaroo care^4^ and breastfeeding^41^ are recognised as effective comfort measures to relieve pain in neonates. Our primary objective was to test whether gentle parental touch, delivered at a speed that optimally activates CT fibres, effectively reduces acute procedural pain in neonates. In the intervention group, parents provided gentle touch before a clinically-required heel lance, while in the control group they provided the same tactile stimulation after the heel lance. The magnitude of noxious-evoked brain activity, the clinical pain score and the occurrence of tachycardia following the heel lance did not significantly differ between groups, and nor did levels of parental anxiety.

The impact of touch on neonatal physiology has been extensively studied.^3^ Our results differ from existing evidence showing that maternal touch, in the form of skin-to-skin contact during clinical procedures, has a positive effect on pain-related brain-derived outcomes.^8,9,11^ Skin-to-skin care includes multiple sensory components, such as parental smell^42^ and hearing soothing voices.^43^ However, a critical aspect of skin-to-skin care is the dynamic tactile interaction between parent and child, that likely activates CT-fibres^20^ and contributes to the intervention efficacy. While this study did not find evidence that parentally-delivered gentle touch reduces neonatal pain, it is important to interpret the data cautiously.

Our design was chosen to match the experimental protocol used in two pilot investigations, where tactile stimulation elicited a substantial reduction in noxious-evoked brain activity.^22,23^ Given the value of involving parents in their baby’s pain management,^44^ rather than applying a researcher-led stroking intervention using a calibrated brush, we asked parents to use their hand to stroke their baby’s skin, which had the additional benefit that CT fibres respond optimally when stimulation is applied at skin temperature^15^. Our approach may have masked the pain-relieving effects of the parent-led intervention through two interlinked factors.

Firstly, it was not feasible for parents to deliver the intervention with the same degree of accuracy and precision as trained researchers in the pilot studies. In the pilot investigations,^22,23^ researchers used a calibrated brush to provide the tactile stimulation with a precise timing immediately before the heel lance as per the experimental design methodology. However, in the intervention group in the Petal trial, there was a median delay of 16·9 s (IQR 11·6–33·0, n=39) between the parental stroking and the heel lance. Increasing the delay between CT-fibre stimulation and the noxious procedure up to as little as five seconds significantly reduces the analgesic efficacy of the intervention^17^ – in retrospect it may have been better to allow parents to stroke their baby for a longer duration, including throughout the heel lance and during the blood collection.

This links to another study limitation and a potential conflict in data interpretation. We guided parents to provide a brief 10-second stroking intervention by following a computer animation that displayed the speed and direction of the strokes; in doing so, we inadvertently created a less natural setting for parents who would usually comfort their babies more intuitively. Consequently, parents’ movements become more mechanical and task-oriented, potentially detracting from the natural parent-infant bonding. Taken together, these two factors may have led to parents providing a less natural form of social touch, which potentially aroused their baby without optimising the CT-fibre mediated pain relief. A more spontaneous approach to delivering the gentle touch, such as allowing parents to stroke their child at their own pace, for as long as they need to calm and comfort their child would likely have been more effective in a clinical setting. In summary, the study design used here inadvertently neither optimised the CT-fibre activation nor optimised parents’ intuitive ability to soothe their baby – this understanding can be used to shape future trial designs.

Asking parents to stroke their babies at their own pace for a longer period of time is strongly supported by a recent study investigating intuitive maternal stroking in preterm babies.^21^ The benefits of parental stroking on neonatal physiology have been demonstrated when parents stroke their baby for a few minutes,^21^ rather than a few seconds as directed in the Petal trial. It is likely that, even without overt direction, parents stroke their babies at CT-optimal speeds, which is observed across species, and naturally in parent-baby interactions.^21,45^ Evidence suggests that when un-directed, parents in a naturalistic setting instinctively alter the delivery of stroking to their child in a context-dependent manner, compared with stroking an inanimate object.^46^ An important next step is to refine the trial design to use a more natural longer form of self-led parental touch.

The Petal trial provides a framework for conducting randomised controlled studies that can objectively assess the impact of parent-led strategies to reduce neonatal pain. A key limitation that needs to be overcome is the high degree of data loss due to data quality affected by noise. Refining the methodology to reduce data loss is a key research goal, as this is a known challenge, particularly in pain studies that rely on single-event recordings. Refined methodology, using an inverse-variance weighted least squares approach, should reduce data loss to less than 5%, which is typically considered acceptable in RCTs.^47^ Nevertheless, post-hoc consideration as part of a methods refinement analysis does not indicate that reducing data loss in this trial would change the core findings. We demonstrated the feasibility and parental acceptability of recording and analysing noxious-evoked brain activity in a multicentre trial, and the high value parents place on this research; more than 70% of eligible families (35% fathers and 65% mothers) gave consent for their babies to take part in the Petal trial, and 98% (104 of 106) of parents who participated in the trial would consider taking part in future studies.^27^ Further work should incorporate intuitive dynamic tactile stroking^21^ into kangaroo care, which has been demonstrated to be effective in providing pain relief.^4^ Acknowledging the challenges faced when translating an experimental approach into a parent-led trial design within the context of a clinical trial is fundamental if progress is to be made establishing the scientific basis that underpins how parents can best support their babies during painful procedures.

While the Petal trial did not show a difference in noxious-evoked brain activity, pain-related behaviour or prevalence of tachycardia following an experimental parental touch intervention, it supports the importance of involving parents in the care of their babies during painful procedures. Performing randomised controlled trials in the neonatal population is a challenging but essential component of evidence-based medicine. The Petal trial highlights the importance of not over-interpreting the clinical relevance of data in small observational studies, which are known to have higher risk of bias compared with pre-registered, randomised, blinded clinical trials. Nevertheless, it is equally important to note that while we did not demonstrate that parental stroking reduced neonatal pain, we cannot rule out the possibility of an effect, as failing to reject the null hypothesis that parental stroking is ineffective, is not equivalent to proving the null hypothesis that the stroking intervention is ineffective. Future RCTs will be required, and appropriately pooling evidence across multiple high-quality trials using meta-analysis will help determine intervention efficacy more conclusively.

## Supplementary Material

Appendix

## Figures and Tables

**Table 1:** Baseline characteristics of all randomised neonates. Data are median (IQR) or count (%). ^*^For the four withdrawn neonates, the parent who had planned to perform the stroking is indicated. Sex was determined based on information provided in the medical notes. The estimated cumulative prior pain exposure indicates skin-breaking blood tests, oral and endotracheal suctions.

Baseline characteristic		Stroking pre-procedure (n = 56)	Stroking post-procedure (n = 56)
Parent stroking*			
	Biological father	20 (36%)	19 (34%)
	Biological mother	36 (64%)	37 (66%)
Gestational age at birth (weeks)		38.8 (36.9–40.1)	38.0 (36.7–39.4)
Postmenstrual age at time of study (weeks)		38.9 (37.2–40.5)	38.4 (37.2–40.1)
Postnatal age at time of study (days)		3 (1–5)	3 (1–5)
Birthweight (g)		3423(2765–3817)	3230 (2770–3722)
Sex			
	Female	22 (39%)	22 (39%)
	Male	34 (61%)	34 (61%)
Mode of delivery			
	Normal vaginal	23 (41%)	20 (36%)
	Breech vaginal	1 (2%)	0
	Elective C-Section	14 (25%)	9 (16%)
	Emergency C-Section	12 (21%)	15 (27%)
	Ventouse/forceps	6 (11%)	12 (21%)
Apgar score at 1 min		9 (7–10)	9 (8–10)
Apgar score at 5 min		10 (9–10)	10 (9–10)
Primary reason for blood test			
	Glucose monitoring	3 (5%)	3 (5%)
	Jaundice	26 (46%)	28 (50%)
	Newborn screening	4 (7%)	4 (7%)
	Suspected sepsis	17 (30%)	16 (29%)
	Other	6 (11%)	5 (9%)
Behavioural state at baseline prior to the heel lance			
	Awake	15 (27%)	15 (27%)
	Asleep	38 (68%)	39 (70%)
	N/A	3 (5%)	2 (4%)
Site			
	Exeter	15 (27%)	14 (25%)
	Oxford	41 (73%)	42 (75%)
Estimated cumulative prior pain exposure		4 (2–6)	4 (2–6)

## Data Availability

The data that support the findings of this study are available from the PI after deidentification (rebeccah.slater@paediatrics.ox.ac.uk). Data will be available after article publication. Data will be shared with investigators whose proposed use of the data has been approved by the PI Professor Rebeccah Slater, to achieve aims in the approved research proposal. The code used to generate the results of the group comparisons for primary and secondary outcomes reported in the manuscript, as well as to generate Figure 2B and Figure 3, is available (https://zenodo.org/record/8429864). The full study protocol is available on ISRCTN (identifier ISRCTN14135962), the SAP is accessible online,^37^ and sample participant information leaflets and informed consent forms are appended to the trial protocol.^28^

## Research in context

### Evidence before this study

Hospitalised neonates receiving medical care frequently require painful clinical procedures. Managing pain presents a considerable challenge, mainly due to the complexity of assessing the effectiveness of pain-relieving interventions in this pre-verbal population. Multiple non-pharmacological approaches, such as skin-to-skin care, baby massage and swaddling are reported to reduce neonatal pain, but the mechanisms underpinning the analgesic efficacy of these touch-based interventions are not well understood. In two independent pilot studies, a trained researcher stroked babies’ skin with a calibrated brush at approximately 3 cm/s prior to a painful procedure. Stroking at this speed can activate C-tactile (CT) afferents, and lead to a reduction in verbal pain scores in adults. Similarly, in both pilot studies we observed a reduction in neonates’ pain-related brain activity.

The intention of the Petal trial was for the stroking intervention to be delivered by parents, therefore a literature search was conducted to identify the impact of parental touch on brain-derived indicators of neonatal pain. On 12 September 2023, we searched MEDLINE, Embase, and the Cochrane Central Register of Controlled Trials (CENTRAL) databases (Appendix pp 3-8). We identified three research studies and one study protocol exploring the relationship between parent-led touch interventions and brain-derived indicators of neonatal pain. The three studies (one randomised controlled trial, one prospective crossover and one prospective cross-sectional study) investigated the effect of maternal skin-to-skin care (kangaroo care) prior to or during clinically-required heel lances or venepunctures. The findings of these studies suggest that maternal skin-to-skin care has beneficial effects, such as improved physiological stability, reduced pain scores and reduced brain-derived noxious-evoked responses. A randomised controlled trial registered in 2018 describes a study protocol to investigate the effect of skin-to-skin contact compared with 24% sucrose on noxious-evoked brain activity in premature babies, measured using EEG during a clinically-required heel lance. The results have not been published. None of the studies assessed the experiences of parents providing the gentle touch intervention.

### Added value of this study

We conducted a multicentre, randomised controlled trial to assess whether parental stroking prior to a clinically-required heel lance can provide pain relief to near-term and term neonates during a painful procedure. Neonatal outcome measures were noxious-evoked brain activity (primary), pain scores (secondary), and development of tachycardia (secondary). A secondary outcome assessed parental anxiety before and after the painful procedure. There was no significant difference in the outcome measures regardless of whether the baby was stroked before or after the painful procedure. However, an independent survey of parents who participated in the trial showed that parents found that providing parental touch to their babies during painful procedures was ‘reassuring’ and ‘useful’.

### Implications of all the available evidence

Non-pharmacological touch interventions are recommended in neonatal pain management guidelines. Nevertheless, indications on how, when, where and by whom touch interventions should be delivered are vague. We assessed a potential mechanism through which touch might provide pain relief, namely stroking at the optimal speed to activate CT fibres. Our results do not indicate that parental stroking for 10 s prior to a painful procedure relieved pain.

This multicentre trial was successfully completed at two sites. Neonatal pain was measured using physiological, behavioural and brain-derived outcomes. The study exemplifies how objective brain-derived measures can be used as a primary outcome measure in clinical trials. Performing high-quality multicentre randomised controlled trials in the neonatal population is a challenging but essential component of evidence-based medicine. The carefully designed trial protocol and comprehensive statistical analysis plan (SAP) can be used as a blueprint for future clinical trials of analgesics in the neonatal setting.
