# Supplementary material for: Effect of parental touch on relieving acute procedural pain in neonates and parental anxiety (Petal): a multicentre randomised controlled trial
Source: Lancet Child Adolesc Health. Author manuscript; Available in PMC 2025 Sep 25. (PMC7618171; doi:10.1016/S2352-4642(23)00340-1)
Supplement: Appendix [file EMS208811-supplement-Appendix.docx]

**Supplementary appendix**

Parental Touch Trial (Petal)

Table of Contents

[Evidence before this study search strategy 3](#_Toc152883253)

[Research Question 3](#_Toc152883254)

[Eligibility Criteria 3](#_Toc152883255)

[Database (Interface) 4](#_Toc152883256)

[Search filters 4](#_Toc152883257)

[MEDLINE (Ovid) 4](#_Toc152883258)

[Embase (Ovid) 6](#_Toc152883259)

[CENTRAL (Wiley) 7](#_Toc152883260)

[Figure S1. PRISMA flowchart diagram^1^ of studies included in the “Evidence before this study” panel. 8](#_Toc152883261)

[Supplementary Trial Documents 8](#_Toc152883262)

[Supplementary Methods 9](#_Toc152883263)

[Figure S2. Overview of trial procedures 9](#_Toc152883264)

[EEG Analysis 9](#_Toc152883265)

[Intention-to-treat analysis 10](#_Toc152883266)

[Statistical models 10](#_Toc152883267)

[Risk ratio for the secondary tachycardia outcome 11](#_Toc152883268)

[CONSORT 2010 Reporting checklist for randomised trial^10^ 11](#_Toc152883269)

[Supplementary Results 14](#_Toc152883270)

[Table S2. Baseline characteristics of neonates included in the analysis of the primary outcome (full analysis set). 14](#_Toc152883271)

[Table S3. Baseline characteristics of neonates included in the analysis of the secondary outcomes. 15](#_Toc152883272)

[Figure S3. Magnitudes of noxious-evoked brain activity during background period, sham heel lance and heel lance. 16](#_Toc152883273)

[Figure S4. Secondary outcomes tachycardia and PIPP-R following sham heel lance and heel lance. 17](#_Toc152883274)

[Figure S5: Average non-Woody-filtered EEG waveforms by stimulus type, site and group 18](#_Toc152883275)

[Intention-to-treat analysis 18](#_Toc152883276)

[Risk ratio for the secondary tachycardia outcome 18](#_Toc152883277)

[References 19](#_Toc152883278)

# Evidence before this study search strategy

## Research Question

Does parent-led touch stimulation prior to or during a painful procedure reduces noxious-evoked brain activity in neonates?

PICO Format

| **P** | Human neonates |
| --- | --- |
| **I** | Parent-led touch stimulation prior to or during painful procedures |
| **C** | n/a |
| **O** | Neonate’s brain activity |

## Eligibility Criteria

Inclusion Criteria

| **Population** | Human neonates |
| --- | --- |
| **Intervention** | Parent-led touch stimulation prior to or during painful procedures |
| **Comparator** | n/a |
| **Outcome** | Brain activity measures |
| **Study design** | All primary empirical research study designs |
| **Report characteristics** | All report types |

Exclusion Criteria

| **Population** | Humans pre-natal and older than neonatal age; non-humans |
| --- | --- |
| **Intervention** | Non-touch stimulations, and non-parent-led interventions |
| **Comparator** | n/a |
| **Outcome** | Studies that do not include brain activity outcomes |
| **Study design** | Non-primary research (e.g. reviews) and non-empirical publications (e.g. editorials, opinion pieces) |
| **Report characteristics** | None |

## Database (Interface)

MEDLINE (Ovid)

Embase (Ovid)

Cochrane Central Register of Controlled Trials (Wiley)

## Search filters

For searching our population-of-interest (i.e. neonates), we used the “foetus and baby” search filter, which is an “Age Specific Filter” from the ISSG Search Filters Resource:

<https://sites.google.com/a/york.ac.uk/issg-search-filters-resource/home/age-groups>

This (Ovid Medline) filter is published on the Canadian Health Libraries Association website:

<https://extranet.santecom.qc.ca/wiki/!biblio3s/doku.php?id=concepts:foetus-et-bebe>

We modified this search filter to exclude irrelevant terms related to “foetus” and “pregnancy”. The full original search filter is copied here below, and terms with strikethrough are irrelevant for our search and were thus deleted from our search:

perinatal* ~~OR antepartum OR ante-partum OR intrapartum OR intra-partum~~ OR neonatal* OR neo-natal* OR postnatal* OR post-natal* ~~OR pregnan* OR fetus* OR fetus* OR fetal* OR fetal*~~ OR baby OR babies OR neonate* OR neo-nate* OR newborn* OR new-born* OR infant*).ti,ab. OR infant/ OR infant, newborn/ OR infant, low birth weight/ OR infant, small for gestational age/ OR infant, very low birth weight/ OR infant, extremely low birth weight/ OR infant, postmature/ OR infant, premature/ OR infant, extremely premature/ OR birth weight/

## MEDLINE (Ovid)

1. infant/ or infant, newborn/ or infant, low birth weight/ or infant, small for gestational age/ or infant, very low birth weight/ or infant, extremely low birth weight/ or infant, postmature/ or infant, premature/ or infant, extremely premature/ or birth weight/ or (perinatal* or neonatal* or neo-natal* or postnatal* or post-natal* or baby or babies or neonate* or neo-nate* or newborn* or new-born* or infant*).ti,ab.
2. pain/ or acute pain/ or nociceptive pain/ or pain, postoperative/ or pain, procedural/ or pain perception/ or nociception/ or hyperalgesia/ or pain measurement/ or pain management/ or pain threshold/ or nociceptors/ or analgesia/ or (pain* or nocicept* or noxious* or allodynia* or allo-dynia* or hyperalges* or hyper-alges* or hypoalges* or hypo-alges* or analges*).ti,ab.
3. neuroimaging/ or exp magnetic resonance imaging/ or exp functional neuroimaging/ or exp spectroscopy, near-infrared/ or exp electroencephalography/ or exp magnetoencephalography/ or exp cortical excitability/ or (brain or cortex or cortical or cerebrum or cerebral or magnetic resonance imag* or mri* or fmri* or functional neuroimage* or near-infrared spectroscop* or nirs* or fnirs* or eeg* or electroencephalogra* or electro-encephalogra* or erp* or event-related potential* or event related potential* or magnetoencephalogra* or magneto-encephalogra* or erf* or event-related field* or evoked response*).ti,ab.
4. touch/ or touch perception/ or therapeutic touch/ or massage/ or kangaroo-mother care method/ or (touch* or massag* or yakson* or "kangaroo care" or "skin to skin" or skin-to-skin or stroked or stroking or brush*).ti,ab.
5. exp parents/ or exp parenting/ or exp parent-child relations/ or exp maternal behavior/ or exp paternal behavior/ or (parent* or mother* or maternal* or father* or paternal* or dyad*).ti,ab.
6. 1 and 2 and 3 and 4 and 5
7. (review or "scientific integrity review" or "systematic review" or meta-analysis or editorial).pt. or (systematic review or meta-analys* or editorial).ti.
8. exp animals/ not humans/
9. 7 or 8
10. 6 not 9

## Embase (Ovid)

1. infant/ or baby/ or high risk infant/ or hospitalized infant/ or newborn/ or low birth weight/ or small for date infant/ or very low birth weight/ or extremely low birth weight/ or postmaturity/ or prematurity/ or birth weight/ or high birth weight/ or (perinatal* or neonatal* or neo-natal* or postnatal* or post-natal* or baby or babies or neonate* or neo-nate* or newborn* or new-born* or infant*).ti,ab.
2. pain/ or allodynia/ or mechanical allodynia/ or tactile allodynia/ or thermal allodynia/ or cold allodynia/ or heat allodynia/ or experimental pain/ or hyperalgesia/ or mechanical hyperalgesia/ or opioid induced hyperalgesia/ or thermal hyperalgesia/ or cold hyperalgesia/ or heat hyperalgesia/ or hypoalgesia/ or thermal hypoalgesia/ or inflammatory pain/ or injection pain/ or injection site pain/ or nociceptive pain/ or postoperative pain/ or procedural pain/ or pain assessment/ or behavioral pain scale/ or nociception/ or gate control theory/ or nociceptive stimulation/ or pain threshold/ or heat pain threshold/ or pressure pain threshold/ or pain receptor/ or pain measurement/ or algometry/ or analgesia/ or antinociception/ or epidural analgesia/ or postoperative analgesia/ or (pain* or nocicept* or noxious* or allodynia* or allo-dynia* or hyperalges* or hyper-alges* or hypoalges* or hypo-alges* or analges*).ti,ab.
3. exp neuroimaging/ or exp nuclear magnetic resonance imaging/ or exp near infrared spectroscopy/ or exp electroencephalography/ or exp magnetoencephalography/ or exp brain function/ or exp brain electrophysiology/ or (brain or cortex or cortical or cerebrum or cerebral or magnetic resonance imag* or mri* or fmri* or functional neuroimage* or near-infrared spectroscop* or nirs* or fnirs* or eeg* or electroencephalogra* or electro-encephalogra* or erp* or event-related potential* or event related potential* or magnetoencephalogra* or magneto-encephalogra* or erf* or event-related field* or evoked response*).ti,ab.
4. touch/ or tactile stimulation/ or vibration sense/ or therapeutic touch/ or massage/ or kangaroo care/ or (touch* or massag* or yakson* or "kangaroo care" or "skin to skin" or skin-to-skin or stroked or stroking or brush*).ti,ab.
5. exp parent/ or exp child parent relation/ or (parent* or mother* or maternal* or father* or paternal* or dyad*).ti,ab.
6. 1 and 2 and 3 and 4 and 5
7. (review or editorial or conference abstract).pt. or (systematic review or meta-analys* or editorial).ti.
8. (exp animal/ or nonhuman/) not exp human/
9. 7 or 8
10. 6 not 9

## CENTRAL (Wiley)

1. [mh ^infant] OR [mh ^"infant, newborn"] OR [mh ^"infant, low birth weight"] OR [mh ^"infant, small for gestational age"] OR [mh ^"infant, very low birth weight"] OR [mh ^"infant, extremely low birth weight"] OR [mh ^"infant, postmature"] OR [mh ^"infant, premature"] OR [mh ^"infant, extremely premature"] OR [mh ^"birth weight"] OR (perinatal*:ti,ab OR neonatal*:ti,ab OR neo-natal*:ti,ab OR postnatal*:ti,ab OR post-natal*:ti,ab OR baby:ti,ab OR babies:ti,ab OR neonate*:ti,ab OR neo-nate*:ti,ab OR newborn*:ti,ab OR new-born*:ti,ab OR infant*:ti,ab)
2. [mh ^pain] OR [mh ^"acute pain"] OR [mh ^"nociceptive pain"] OR [mh ^"pain, postoperative"] OR [mh ^"pain, procedural"] OR [mh ^"pain perception"] OR [mh ^nociception] OR [mh ^hyperalgesia] OR [mh ^"pain measurement"] OR [mh ^"pain management"] OR [mh ^"pain threshold"] OR [mh ^nociceptors] OR [mh ^analgesia] OR (pain*:ti,ab OR nocicept*:ti,ab OR noxious*:ti,ab OR allodynia*:ti,ab OR allo-dynia*:ti,ab OR hyperalges*:ti,ab OR hyper-alges*:ti,ab OR hypoalges*:ti,ab OR hypo-alges*:ti,ab OR analges*:ti,ab)
3. [mh ^neuroimaging] OR [mh "magnetic resonance imaging"] OR [mh "functional neuroimaging"] OR [mh "spectroscopy, near-infrared"] OR [mh electroencephalography] OR [mh magnetoencephalography] OR [mh "cortical excitability"] OR (brain:ti,ab OR cortex:ti,ab OR cortical:ti,ab OR cerebrum:ti,ab OR cerebral:ti,ab OR ("magnetic resonance" NEXT imag*):ti,ab OR mri*:ti,ab OR fmri*:ti,ab OR ("functional" NEXT neuroimage*):ti,ab OR ("near-infrared" NEXT spectroscop*):ti,ab OR nirs*:ti,ab OR fnirs*:ti,ab OR eeg*:ti,ab OR electroencephalogra*:ti,ab OR electro-encephalogra*:ti,ab OR erp*:ti,ab OR ("event-related" NEXT potential*):ti,ab OR ("event related" NEXT potential*):ti,ab OR magnetoencephalogra*:ti,ab OR magneto-encephalogra*:ti,ab OR erf*:ti,ab OR ("event-related" NEXT field*):ti,ab OR ("evoked" NEXT response*):ti,ab)
4. [mh ^touch] OR [mh ^"touch perception"] OR [mh ^"therapeutic touch"] OR [mh ^massage] OR [mh ^"kangaroo-mother care method"] OR (touch*:ti,ab OR massag*:ti,ab OR yakson*:ti,ab OR "kangaroo care":ti,ab OR "skin to skin":ti,ab OR skin-to-skin:ti,ab OR stroked:ti,ab OR stroking:ti,ab OR brush*:ti,ab)
5. [mh parents] OR [mh parenting] OR [mh "parent-child relations"] OR [mh "maternal behavior"] OR [mh "paternal behavior"] OR (parent*:ti,ab OR mother*:ti,ab OR maternal*:ti,ab OR father*:ti,ab OR paternal*:ti,ab OR dyad*:ti,ab)
6. #1 AND #2 AND #3 AND #4 AND #5

## Figure S1. PRISMA flowchart diagram^1^ of studies included in the “Evidence before this study” panel.

*The PRISMA flowchart details the results from the literature search, screening and included studies.*

# Supplementary Trial Documents

- Full Trial Protocol v5·0 ([available on ISRCTN, identifier ISRCTN14135962](https://www.isrctn.com/ISRCTN14135962))
- PIL Exeter and Oxford ([available as Supplement of published protocol](https://bmjopen.bmj.com/content/bmjopen/suppl/2022/07/19/bmjopen-2022-061841.DC1/bmjopen-2022-061841supp001_data_supplement.pdf))^2^
- CF Exeter and Oxford ([available as Supplement of published protocol](https://bmjopen.bmj.com/content/bmjopen/suppl/2022/07/19/bmjopen-2022-061841.DC1/bmjopen-2022-061841supp001_data_supplement.pdf))^2^

# Supplementary Methods

## Figure S2. Overview of trial procedures


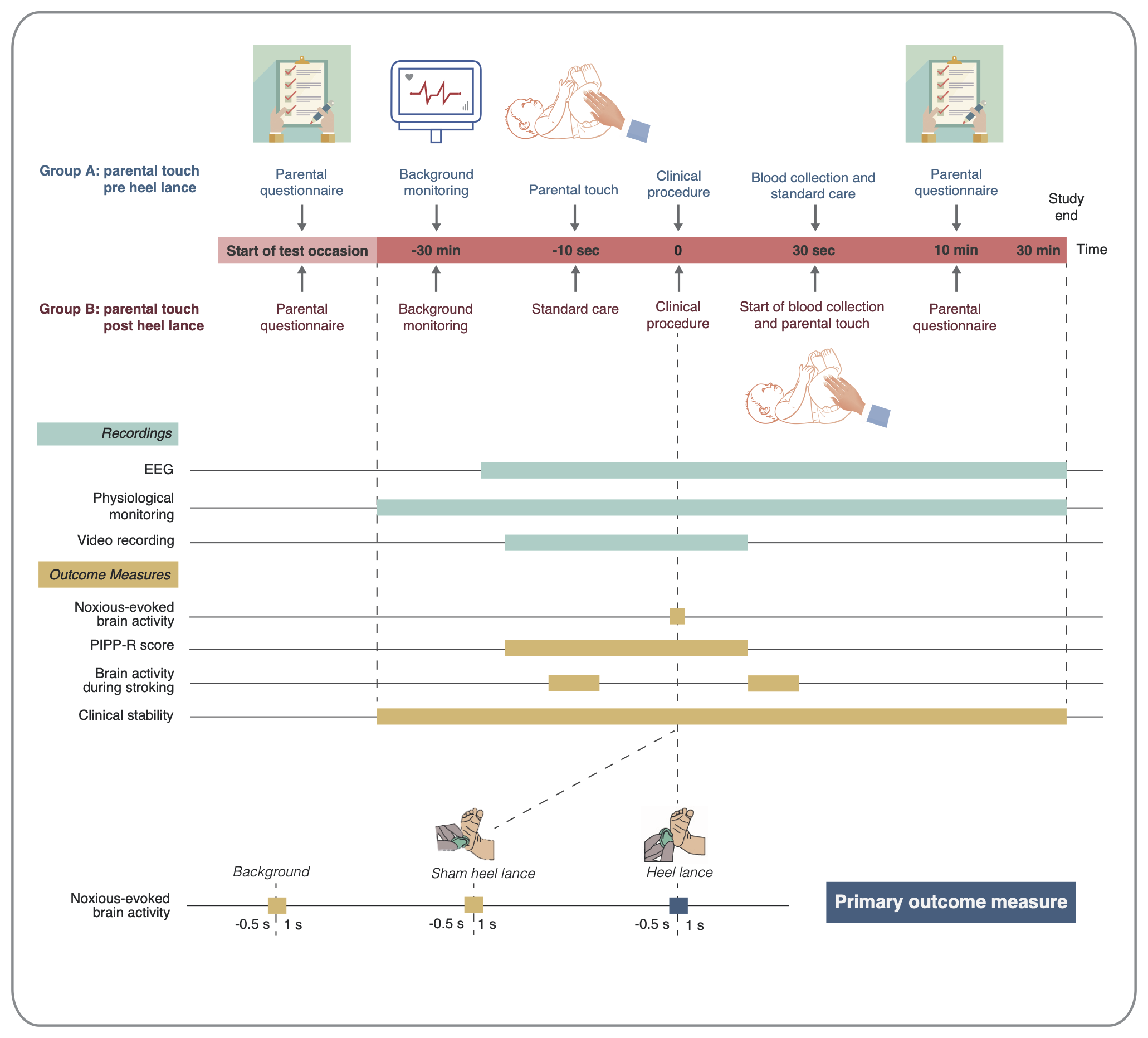


*Petal trial test occasion timeline.* *Overview of trial procedures for the intervention and control groups. Multimodal recordings and neonatal outcome measures. EEG, electroencephalography; PIPP-R, Premature Infant Pain Profile-Revised.*

## EEG Analysis

EEG data were analysed using EEGlab.^3^ Raw data were downsampled to 500 Hz and filtered from 0·5—33·75 Hz using Hamming-windowed sinc FIR filters (high-pass -6 dB cut-off = 0·5 Hz, transition band width = 1 Hz, low-pass -6 dB cut-off = 33·75 Hz, transition band width = 7·5 Hz). Data at the Cz electrode with reference at Fz were epoched around the heel lance and control heel lance with 500 ms before and 1500 ms after the stimulus. Epochs were baseline-corrected by subtracting the mean in the 500 ms pre-stimulus. Epochs were Woody-filtered to align them to the pre-defined neurodynamic response function (n-NRF)^4^ with a maximum jitter of ± 100 ms in the region of 400—700 ms after the stimulus. This involves finding the maximum cross-correlation of the n-NRF with the data.^4^ The n-NRF was then projected onto each individual trial in the region 400—700 ms after the stimulus to calculate the magnitude of the noxious-evoked brain activity. The projection is performed by linear regression, where the regression coefficient represents the magnitude of the noxious-evoked brain activity. Individual EEG traces were rejected if the point of stimulation was not marked on the EEG recording due to technical failure or if there was artefact (for example, gross movement artefact, electrical interference) during the immediate baseline or post-stimulus period.

## Intention-to-treat analysis

This exploratory analysis assessed the intention-to-treat (ITT) effect, i.e., the effect of being assigned to the intervention. The ITT analysis was performed on the full analysis set. The ITT analysis assessed the effect of the group assigned at randomisation, regardless of any subsequent non-compliance, on the outcome measures. In contrast, the main analysis presented in the manuscript assessed the effect of adherence to the intervention (per protocol effect).

The primary outcome (magnitude of the n-NRF) and the secondary outcomes Premature Infant Pain Profile-Revised (PIPP-R) and State Trait Anxiety Inventory-State (STAI-S) were compared between the intervention and control groups using multiple linear regression analysis. The development of tachycardia (binary secondary outcome) was compared between the groups using a logistic regression. In the regression models, the variable of group allocation at randomisation was adjusted for the five minimisation variables (gestational age, postnatal age, site, sex and primary reason for blood test). In the analysis of the STAI-S outcome, the group allocation variable was additionally adjusted for the STAI-S at baseline (i.e., before the heel lance).

## Statistical models

In line with our statistical analysis plan,^5^ we assessed the distribution of residuals to test assumptions underlying the statistical models. Residuals of the three linear regression models (EEG, PIPP-R, and STAI-S outcomes) were not normally distributed, so non-parametric p-values derived using permutation testing are reported. Of the three linear regression analyses, residuals were heteroscedastic for both EEG and STAI-S outcomes, so robust linear regression was used. While the PIPP-R outcome had homoscedastic residuals, for consistency robust linear regression was used for the PIPP-R analysis. However, using ordinary least squares (OLS) linear regression for PIPP-R data produces results very closely matched the robust analysis, with slightly larger effect sizes identified using robust regression.

We tested the linearity assumption for the linear models with multivariable fractional polynomials (FPs) using the *mfp* package in R.^6,7^ This algorithm identifies the most appropriate functional form for continuous predictors in a linear model. We fitted linear regressions for the EEG, PIPP-R, and STAI-S outcomes and a logistic regression for the tachycardia outcome. Gestational age (GA), postnatal age (PNA), sex, site and the reason for blood test were included in all models as covariates. In the STAI-S model, we also included baseline STAI-S as a covariate. In brief, for each continuous predictor in turn (GA, PNA and baseline STAI-S), the mfp algorithm estimates the most appropriate functional form in an iterative way, which includes testing the FPs against a straight line. The p-value for this comparison is based on the difference in deviances between the FP models and the linear model. This test was non-significant for all the regression models and all continuous variables (Table S1), thus confirming the linearity assumption.

**Table S1. P-values for the test of linearity for the continuous predictors in each of the four regression models, as obtained by the multivariate fractional polynomials algorithm.** GA = gestational age at birth in weeks. PNA = postnatal days at study.

| ***Predictors***  ***Outcomes*** | **GA** | **PNA** | **Baseline STAI-S** |
| --- | --- | --- | --- |
| **EEG** | 0*·*87 | 0*·*29 | N/A |
| **PIPP-R** | 0*·*79 | 0*·*97 | N/A |
| **Tachycardia** | 0*·*99 | 1*·*00 | N/A |
| **STAI-S** | 0*·*62 | 0*·*95 | 0*·*87 |

## Risk ratio for the secondary tachycardia outcome

The development of tachycardia (binary secondary outcome) was compared between the groups using a robust Poisson regression to allow reporting of risk ratio outcomes.^8^ This was implemented in R using the sandwich package.^9^

## CONSORT 2010 Reporting checklist for randomised trial^10^

|  |  | Reporting Item | Page Number |
| --- | --- | --- | --- |
| **Title and Abstract** |  |  |  |
| Title | [#1a](https://www.goodreports.org/reporting-checklists/consort/info/#1a) | Identification as a randomized trial in the title. | 1 |
| Abstract | [#1b](https://www.goodreports.org/reporting-checklists/consort/info/#1b) | Structured summary of trial design, methods, results, and conclusions |  |
| **Introduction** |  |  |  |
| Background and objectives | [#2a](https://www.goodreports.org/reporting-checklists/consort/info/#2a) | Scientific background and explanation of rationale | 3-4 |
| Background and objectives | [#2b](https://www.goodreports.org/reporting-checklists/consort/info/#2b) | Specific objectives or hypothesis | 4 |
| **Methods** |  |  |  |
| Trial design | [#3a](https://www.goodreports.org/reporting-checklists/consort/info/#3a) | Description of trial design (such as parallel, factorial) including allocation ratio. | 5 |
| Trial design | [#3b](https://www.goodreports.org/reporting-checklists/consort/info/#3b) | Important changes to methods after trial commencement (such as eligibility criteria), with reasons | n/a |
| Participants | [#4a](https://www.goodreports.org/reporting-checklists/consort/info/#4a) | Eligibility criteria for participants | 5 |
| Participants | [#4b](https://www.goodreports.org/reporting-checklists/consort/info/#4b) | Settings and locations where the data were collected | 5 |
| Interventions | [#5](https://www.goodreports.org/reporting-checklists/consort/info/#5) | The experimental and control interventions for each group with sufficient details to allow replication, including how and when they were actually administered | 5 |
| Outcomes | [#6a](https://www.goodreports.org/reporting-checklists/consort/info/#6a) | Completely defined prespecified primary and secondary outcome measures, including how and when they were assessed | 7, SAP |
| Outcomes | [#6b](https://www.goodreports.org/reporting-checklists/consort/info/#6b) | Any changes to trial outcomes after the trial commenced, with reasons | n/a |
| Sample size | [#7a](https://www.goodreports.org/reporting-checklists/consort/info/#7a) | How sample size was determined. | 7 |
| Sample size | [#7b](https://www.goodreports.org/reporting-checklists/consort/info/#7b) | When applicable, explanation of any interim analyses and stopping guidelines | n/a |
| Randomization - Sequence generation | [#8a](https://www.goodreports.org/reporting-checklists/consort/info/#8a) | Method used to generate the random allocation sequence. | 5 |
| Randomization - Sequence generation | [#8b](https://www.goodreports.org/reporting-checklists/consort/info/#8b) | Type of randomization; details of any restriction (such as blocking and block size) | 5 |
| Randomization - Allocation concealment mechanism | [#9](https://www.goodreports.org/reporting-checklists/consort/info/#9) | Mechanism used to implement the random allocation sequence (such as sequentially numbered containers), describing any steps taken to conceal the sequence until interventions were assigned | 5 |
| Randomization - Implementation | [#10](https://www.goodreports.org/reporting-checklists/consort/info/#10) | Who generated the allocation sequence, who enrolled participants, and who assigned participants to interventions | 5 |
| Blinding | [#11a](https://www.goodreports.org/reporting-checklists/consort/info/#11a) | If done, who was blinded after assignment to interventions (for example, participants, care providers, those assessing outcomes) and how. | 6 |
| Blinding | [#11b](https://www.goodreports.org/reporting-checklists/consort/info/#11b) | If relevant, description of the similarity of interventions | n/a |
| Statistical methods | [#12a](https://www.goodreports.org/reporting-checklists/consort/info/#12a) | Statistical methods used to compare groups for primary and secondary outcomes | 7-8 |
| Statistical methods | [#12b](https://www.goodreports.org/reporting-checklists/consort/info/#12b) | Methods for additional analyses, such as subgroup analyses and adjusted analyses | 7-8 |
| **Results** |  |  |  |
| Participant flow diagram (strongly recommended) | [#13a](https://www.goodreports.org/reporting-checklists/consort/info/#13a) | For each group, the numbers of participants who were randomly assigned, received intended treatment, and were analysed for the primary outcome | 10 |
| Participant flow | [#13b](https://www.goodreports.org/reporting-checklists/consort/info/#13b) | For each group, losses and exclusions after randomization, together with reason | 10 |
| Recruitment | [#14a](https://www.goodreports.org/reporting-checklists/consort/info/#14a) | Dates defining the periods of recruitment and follow-up | 9 |
| Recruitment | [#14b](https://www.goodreports.org/reporting-checklists/consort/info/#14b) | Why the trial ended or was stopped | n/a |
| Baseline data | [#15](https://www.goodreports.org/reporting-checklists/consort/info/#15) | A table showing baseline demographic and clinical characteristics for each group | 11 |
| Numbers analysed | [#16](https://www.goodreports.org/reporting-checklists/consort/info/#16) | For each group, number of participants (denominator) included in each analysis and whether the analysis was by original assigned groups | 12-13 |
| Outcomes and estimation | [#17a](https://www.goodreports.org/reporting-checklists/consort/info/#17a) | For each primary and secondary outcome, results for each group, and the estimated effect size and its precision (such as 95% confidence interval) | 12-13 |
| Outcomes and estimation | [#17b](https://www.goodreports.org/reporting-checklists/consort/info/#17b) | For binary outcomes, presentation of both absolute and relative effect sizes is recommended | 12-13 |
| Ancillary analyses | [#18](https://www.goodreports.org/reporting-checklists/consort/info/#18) | Results of any other analyses performed, including subgroup analyses and adjusted analyses, distinguishing pre-specified from exploratory | 11, appendix |
| Harms | [#19](https://www.goodreports.org/reporting-checklists/consort/info/#19) | All important harms or unintended effects in each group (For specific guidance see CONSORT for harms) | 11 |
| **Discussion** |  |  |  |
| Limitations | [#20](https://www.goodreports.org/reporting-checklists/consort/info/#20) | Trial limitations, addressing sources of potential bias, imprecision, and, if relevant, multiplicity of analyses | 14 |
| Generalisability | [#21](https://www.goodreports.org/reporting-checklists/consort/info/#21) | Generalisability (external validity, applicability) of the trial findings | 15 |
| Interpretation | [#22](https://www.goodreports.org/reporting-checklists/consort/info/#22) | Interpretation consistent with results, balancing benefits and harms, and considering other relevant evidence | 14-15 |
| Registration | [#23](https://www.goodreports.org/reporting-checklists/consort/info/#23) | Registration number and name of trial registry | 9 |
| **Other information** |  |  |  |
| Interpretation | [#22](https://www.goodreports.org/reporting-checklists/consort/info/#22) | Interpretation consistent with results, balancing benefits and harms, and considering other relevant evidence | 14-15 |
| Registration | [#23](https://www.goodreports.org/reporting-checklists/consort/info/#23) | Registration number and name of trial registry | 9 |
| Protocol | [#24](https://www.goodreports.org/reporting-checklists/consort/info/#24) | Where the full trial protocol can be accessed, if available | appendix |
| Funding | [#25](https://www.goodreports.org/reporting-checklists/consort/info/#25) | Sources of funding and other support (such as supply of drugs), role of funders | 17 |

# Supplementary Results

Table S2. Baseline characteristics of neonates included in the analysis of the primary outcome (full analysis set). **Median (interquartile range (IQR)) or count (%) are reported.**

| **Baseline characteristics** | **EEG** | |
| --- | --- | --- |
|  | **Intervention**  **(n=39)** | **Control**  **(n=43)** |
| Parent stroking   - Biological father - Biological mother | 12 (31%)  27 (69%) | 14 (33%)  29 (67%) |
| Gestational age at birth (weeks) | 38·1 (36·9–40·0) | 38·1 (36·7–39·4) |
| Postmenstrual age at time of study (weeks) | 38·3 (37·1–40·4) | 38·3 (37·2–39·9) |
| Postnatal age at time of study (days) | 2 (1–5) | 3 (1–5) |
| Birthweight (g) | 3385 (2696–3757) | 3225 (2690–3762) |
| Sex   - Female - Male | 14 (36%)  25 (64%) | 18 (42%)  25 (58%) |
| Mode of delivery   - Normal vaginal - Breech vaginal - Elective C-Section - Emergency C-Section - Ventouse/forceps | 16 (41%)  1 (3%)  10 (26%)  8 (20%)  4 (10%) | 16 (37%)  0  5 (12%)  13 (30%)  9 (21%) |
| Apgar score at 1 min | 9 (7–10) | 9 (8–10) |
| Apgar score at 5 min | 10 (9–10) | 10 (9–10) |
| Primary reason for blood test   - Glucose monitoring - Jaundice - Newborn screening - Suspected sepsis - Other | 2 (5%)  17 (44%)  3 (8%)  13 (33%)  4 (10%) | 3 (7%)  22 (51%)  3 (7%)  13 (30%)  2 (5%) |
| Site   - Exeter - Oxford | 13 (33%)  26 (67%) | 14 (33%)  29 (67%) |
| Estimated cumulative prior pain exposure | 4 (2–6) | 4 (2–6) |
| Time (s) between stroking start (pre-heel lance) and heel lance | 16·9 (11·6–33·0) | N/A |
| Time (s) between heel lance and stroking start (post-heel lance) | N/A | 46·5 (42·1–106·3) |

Table S3. Baseline characteristics of neonates included in the analysis of the secondary outcomes. **Median (IQR) or count (%) are reported.**

| **Baseline characteristics** | **PIPP-R** | | **Tachycardia** | | **STAI-S** | |
| --- | --- | --- | --- | --- | --- | --- |
|  | **Intervention**  **(n=49)** | **Control**  **(n=51)** | **Intervention**  **(n=52)** | **Control**  **(n=53)** | **Intervention**  **(n=54)** | **Control**  **(n=52)** |
| Parent stroking   - Biological father - Biological mother | 17 (35%)  32 (65%) | 15 (29%)  36 (71%) | 18 (35%)  34 (65%) | 17 (32%)  36 (68%) | 19 (35%)  35 (65%) | 17 (33%)  35 (67%) |
| Gestational age at birth (weeks) | 38·7 (36·9–40·0) | 38·1 (36·7–39·4) | 38·8 (36·9–40·0) | 38·1 (36·7–39·4) | 38·8 (36·9–40·0) | 38·1 (36·7–39·6) |
| Postmenstrual age at time of study (weeks) | 38·7 (37·3–40·3) | 38·6 (37·3–40·2) | 38·9 (37·3–40·4) | 38·6 (37·3–40·1) | 38·9 (37·3–40·4) | 38·6 (37·2–40·3) |
| Postnatal age at time of study (days) | 2 (1–5) | 3 (1–5) | 3 (1–5) | 3 (1–5) | 3 (1–5) | 3 (1–5) |
| Birthweight (g) | 3390 (2740–3765) | 3240 (2745–3762) | 3387 (2740–3755) | 3235 (2710–3737) | 3392 (2740–3760) | 3237 (2700–3750) |
| Sex   - Female - Male | 18 (37%)  31 (63%) | 19 (37%)  32 (63%) | 20 (38%)  32 (62%) | 21 (40%)  32 (60%) | 21 (39%)  33 (61%) | 20 (38%)  32 (62%) |
| Mode of delivery   - Normal vaginal - Breech vaginal - Elective C-Section - Emergency C-Section - Ventouse/forceps | 21 (43%)  1 (2%)  13 (27%)  8 (16%)  6 (12%) | 19 (37%)  0  8 (16%)  13 (25%)  11 (22%) | 22 (42%)  1 (2%)  14 (27%)  9 (17%)  6 (12%) | 20 (38%)  0  8 (15%)  14 (26%)  11 (21%) | 22 (41%)  1 (2%)  14 (26%)  11 (20%)  6 (11%) | 19 (37%)  0  8 (15%)  15 (29%)  10 (19%) |
| Apgar score at 1 min | 9 (8–10) | 9 (8–10) | 9 (7–10) | 9 (8–10) | 9 (8–10) | 9 (8–10) |
| Apgar score at 5 min | 10 (10–10) | 10 (9–10) | 10 (9–10) | 10 (9–10) | 10 (10–10) | 10 (9–10) |
| Primary reason for blood test   - Glucose monitoring - Jaundice - Newborn screening - Suspected sepsis - Other | 2 (4%)  21 (43%)  4 (8%)  17 (35%)  5 (10%) | 3 (6%)  25 (49%)  3 (6%)  15 (29%)  5 (10%) | 2 (4%)  24 (46%)  4 (8%)  17 (33%)  5 (10%) | 3 (6%)  26 (49%)  3 (6%)  16 (30%)  5 (9%) | 2 (4%)  25 (46%)  4 (7%)  17 (32%)  6 (11%) | 3 (6%)  26 (50%)  3 (6%)  15 (29%)  5 (10%) |
| Site   - Exeter - Oxford | 14 (29%)  35 (71%) | 14 (28%)  37 (73%) | 15 (29%)  37 (71%) | 14 (26%)  39 (74%) | 15 (28%)  39 (72%) | 14 (27%)  38 (73%) |
| Estimated cumulative prior pain exposure | 4 (2–6) | 4 (2–7) | 4 (2–6) | 4 (2–6) | 4 (2–6) | 4 (2–6) |
| Time (s) between stroking start (pre-heel lance) and heel lance | 17·3 (12·8–31·7) | N/A | 17·2 (12·6–32·3) | N/A | 17·0 (12·3–31·7) | N/A |
| Time (s) between heel lance and stroking start (post-heel lance) | N/A | 50·8 (42·2–125·2) | N/A | 50·8 (42·2–122·7) | N/A | 49·4 (42·1–117·6) |

## Figure S3. Magnitudes of noxious-evoked brain activity during background period, sham heel lance and heel lance.

*
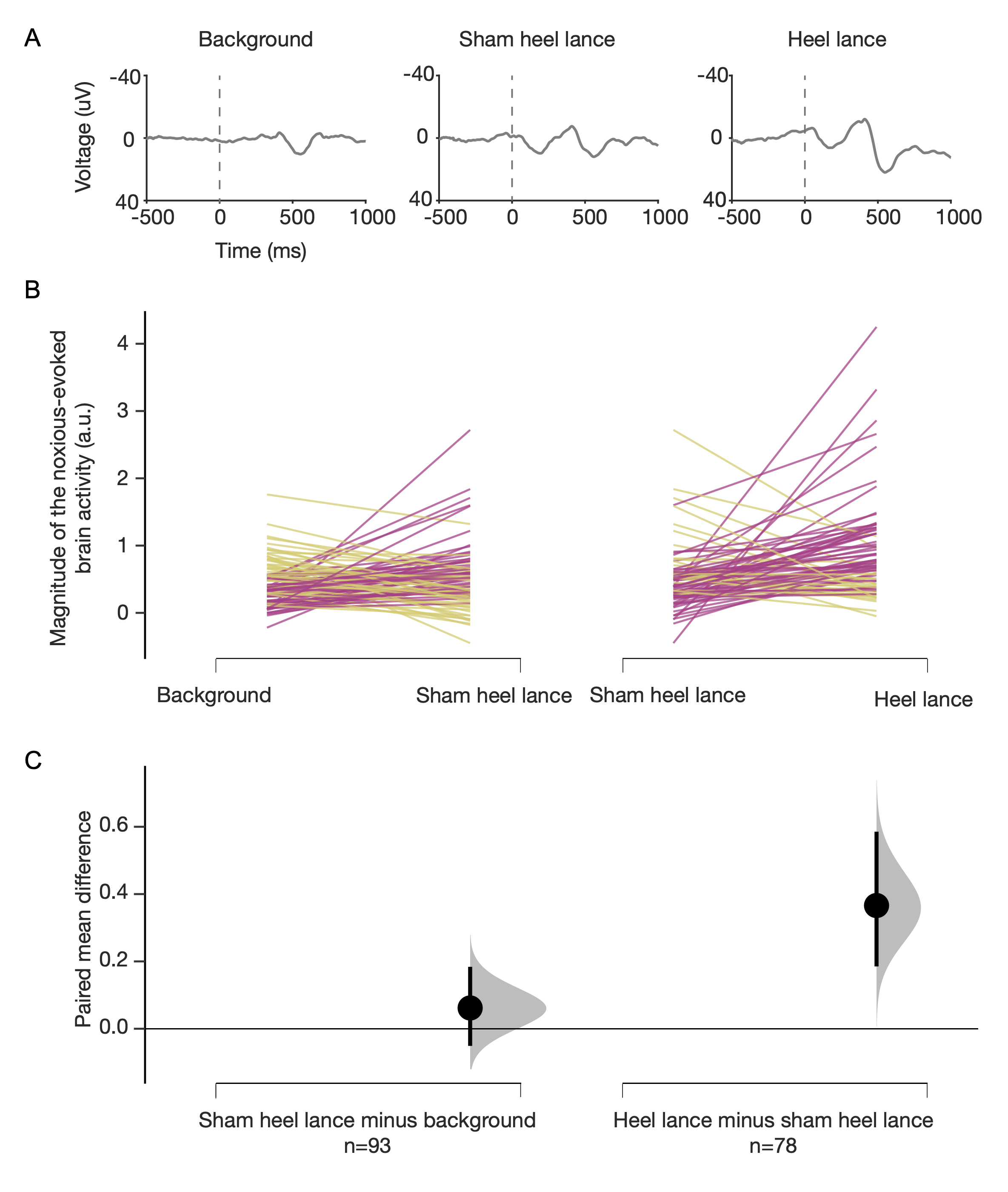
*

*Brain activity during background period, in response to the sham heel lance, and in response to the heel lance. Background EEG epochs were extracted from the period before the sham heel lance. Data from both arms is included. (A) The average EEG traces recorded at electrode Cz during background periods (n=105), as well as from 500 ms preceding to 1000 ms following the sham heel lance (n=94) and heel lance (n=82). EEG data is processed as described in the appendix (pp 9-10) including Woody-filtering to the n-NRF used in this study. (B) Magnitudes of noxious-evoked brain activity as quantified by the n-NRF, paired by participant. The mean (SD) magnitude of the n-NRF was 0·45 (0·34, n=105) during background recordings, 0·51 (0·46, n=94) following the sham heel lance, and 0·88 (0·73, n=82) following the clinically-required heel lance. The lines connecting the magnitudes of the n-NRF indicate whether the neonate had the same or increased magnitude (pink lines), or decreased magnitude (yellow lines) in response to different stimuli. (C) Paired difference of n-NRF magnitudes. The mean n-NRF during the background period and in response to the sham heel lance do not significantly differ (paired t-test, mean difference 0.06, non-parametric p = 0·31, 95% bootstrap confidence interval (CI)=[-0·05,0·18], n=93). Magnitudes of the n-NRF differ significantly between sham heel lance and heel lance responses at Bonferroni corrected alpha level of 0.05/2=0.025 (paired t-test, mean difference 0·37, non-parametric p < 0·001, bootstrap 95% CI=[0·19,0·59], n=78). (B) and (C) were generated using the dabestr package in R.*^11,12^

## Figure S4. Secondary outcomes tachycardia and PIPP-R following sham heel lance and heel lance.

**
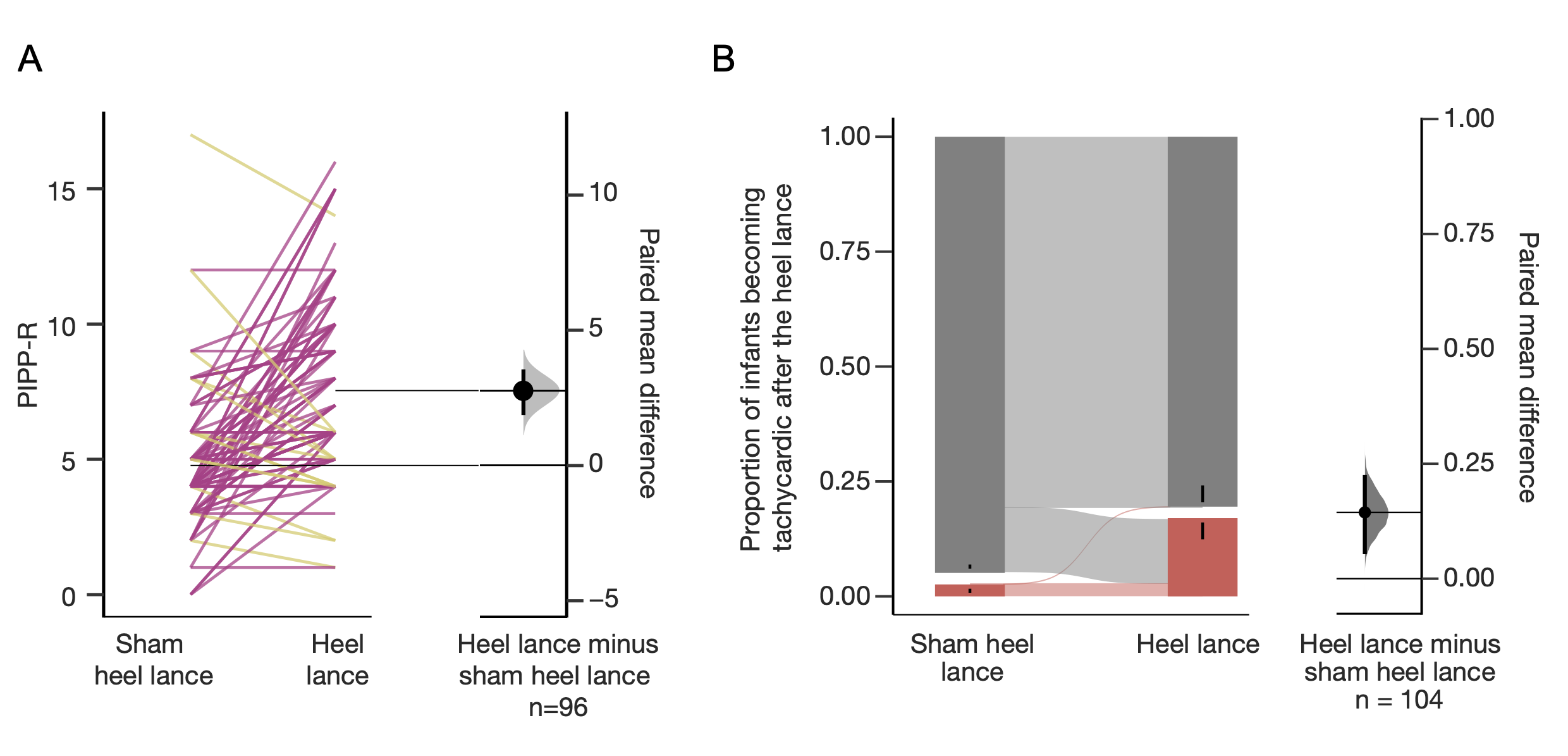
**

*(A) PIPP-R scores in response to the sham heel lance and heel lance, paired by participant. The mean (SD) PIPP-R score following the sham heel lance was 4·81 (2·53, n=98). The mean (SD) PIPP-R score following the heel lance was 7·63 (3·39, n=100). The PIPP-R scores following the sham heel lance and heel lance differ significantly (paired t-test, mean difference 2·77, non-parametric p < 0·001, bootstrap 95% CI=[2·03,3·51], n=96). The lines connecting the PIPP-R score in response to the sham heel lance and heel lance indicate whether the PIPP-R score of the neonate remained the same or increased (pink lines), or decreased (yellow lines) between sham heel lance and heel lance. (B) Development of tachycardia following the sham heel lance and heel lance, paired by participant. Following the sham heel lance 4/105 (4%) neonates developed tachycardia, while following the heel lance 19/105 (18%) developed tachycardia. The occurrence of tachycardia following the sham heel lance and the heel lance differ significantly (difference in proportions 0·14, non-parametric p-value < 0·001, bootstrap 95% CI=[0·06,0·22]; McNemar's chi-squared = 11·53, degrees of freedom (df)=1, n=104). The proportion of infants developing tachycardia is indicated by the red bar. (A) and (B) were generated using the dabestr package in R.*^11,12^

## Figure S5: Average non-Woody-filtered EEG waveforms by stimulus type, site and group


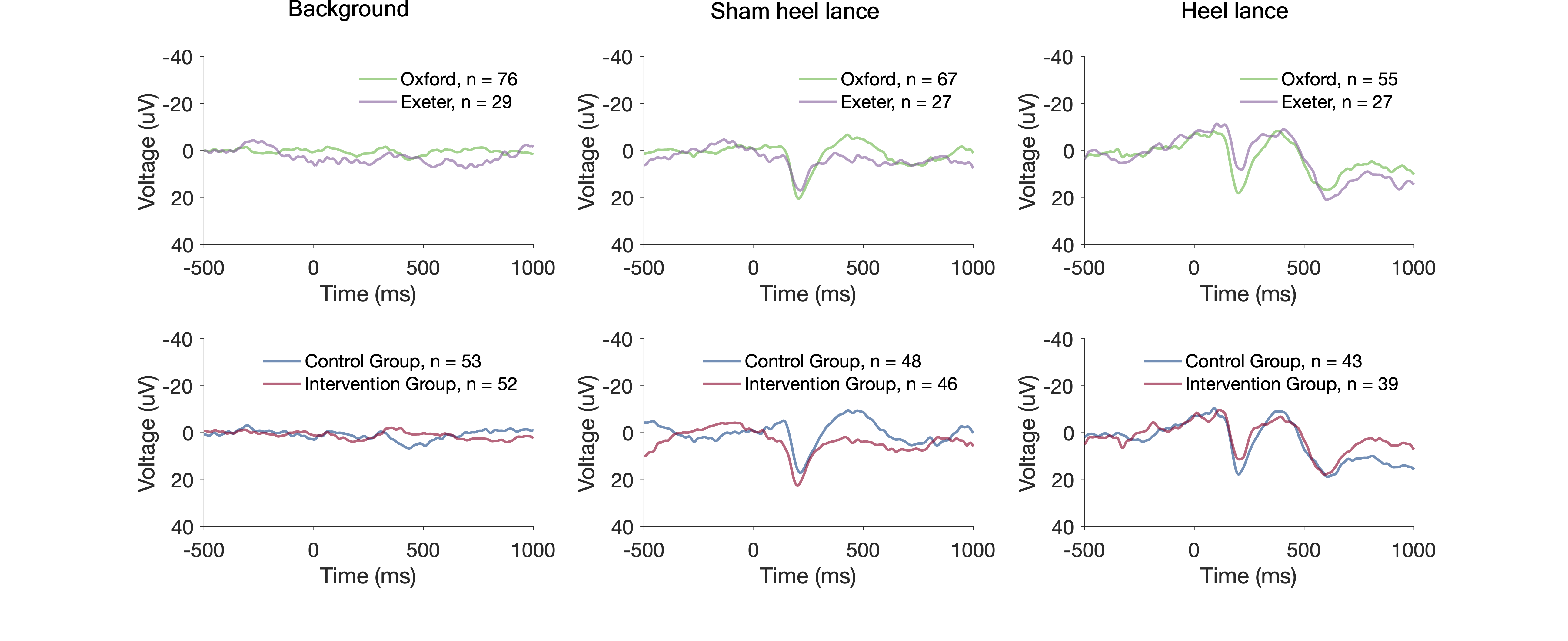


*Average EEG traces in the full analysis set. Raw EEG traces were processed as described in EEG details (appendix), with exception of Woody-filtering. Both the sham heel lance and the heel lance evoke an early positive deflection, as previously described.*^13^ *The characteristic noxious-evoked positive deflection*^4^ *occurring later between 400 and 700 ms is visible in response to the heel lance, but not to the sham heel lance. Waveform morphology is consistent across the two research sites.*

## Intention-to-treat analysis

The ITT was carried out to assess the effect of assignment to the intervention and used the full analysis set. As is expected, the effect of non-compliance resulted in a smaller ITT effect size compared to the per protocol effect size, and was similarly non-significant. Averages and SD of the outcomes are reported below for groupings as randomised.

The primary outcome measure was the magnitude of noxious-evoked brain activity following the heel lance as quantified by the n-NRF. There was no significant difference between groups: difference in means -0·10 (smaller in intervention group), SD 0·77, 95% CI=[-0·37,0·18], p-value 0·37 (n=82).

None of the secondary outcomes differed significantly between intervention and control group. For the PIPP-R outcome, the difference in means between the two groups was 0·94 (greater in intervention group), SD 3·26, 95% CI=[-0·35,2·23], p-value 0·15 (n=100). For the tachycardia outcome, the odds ratio (OR) was 1·86 (greater in the intervention group), 95% CI=[0·52,6·64], p-value 0·34 (n=105). For the parental anxiety STAI-S outcome, the mean difference between groups was -0.44 (greater in control group), SD 6·85, 95% CI=[-2·91, 2·02], p-value 0·72 (n=106).

## Risk ratio for the secondary tachycardia outcome

The risk ratio (RR) was 1·47 (greater in the intervention group), 95% CI was [0·62, 3·48], and p-value 0·38 (n=105).

# References

1 Matthew J Page, Joanne E McKenzie, Patrick M Bossuyt, *et al.* The PRISMA 2020 statement: an updated guideline for reporting systematic reviews. *BMJ* 2021; **372**: n71.

2 Cobo MM, Moultrie F, Hauck AGV, *et al.* Multicentre, randomised controlled trial to investigate the effects of parental touch on relieving acute procedural pain in neonates (Petal). *BMJ Open* 2022; **12**: e061841.

3 Delorme A, Makeig S. EEGLAB: an open source toolbox for analysis of single-trial EEG dynamics including independent component analysis. *J Neurosci Methods* 2004; **134**: 9–21.

4 Hartley C, Duff EP, Green G, *et al.* Nociceptive brain activity as a measure of analgesic efficacy in infants. *Sci Transl Med* 2017; **9**. DOI:10.1126/scitranslmed.aah6122.

5 Baxter L, Hauck AGV, Bhatt A, *et al.* Statistical analysis plan for the Petal trial: the effects of parental touch on relieving acute procedural pain in neonates [version 1; peer review: awaiting peer review]. *Wellcome Open Res* 2023; **8**: 402.

6 Benner A. mfp: Multivariable fractional polynomials. *R News 5(2): 20–23* 2005.

7 Ambler G, Royston P. Fractional polynomial model selection procedures: investigation of type i error rate. *J Stat Comput Simul* 2001; **69**: 89–108.

8 Zou G. A Modified Poisson Regression Approach to Prospective Studies with Binary Data. *Am J Epidemiol* 2004; **159**: 702–6.

9 Zeileis A, Köll S, Graham N. Various Versatile Variances: An Object-Oriented Implementation of Clustered Covariances in R. *J Stat Softw* 2020; **95**: 1–36.

10 Schulz KF, Altman DG, Moher D, Fergusson D. CONSORT 2010 changes and testing blindness in RCTs. *Lancet Br Ed* 2010; **375**: 1144–6.

11 Ho J, Tumkaya T, Aryal S, Choi H, Claridge-Chang A. Moving beyond P values: data analysis with estimation graphics. 2019. DOI:10.1038/s41592-019-0470-3.

12 R Core Team. R: A Language and Environment for Statistical Computing. 2022. https://www.R-project.org/.

13 Slater R, Worley A, Fabrizi L, *et al.* Evoked potentials generated by noxious stimulation in the human infant brain. *Eur J Pain Lond Engl* 2010; **14**: 321–6.
